# Antibacterial Properties and Mechanism of Lysozyme-Modified ZnO Nanoparticles

**DOI:** 10.3389/fchem.2021.762255

**Published:** 2021-11-26

**Authors:** Kangrui Yuan, Xiaoliu Liu, Jianxin Shi, Wei Liu, Kun Liu, Hongmei Lu, Dudu Wu, Zhi Chen, Chengyu Lu

**Affiliations:** ^1^ School of Pharmacy, Guangdong Medical University, Dongguan, China; ^2^ Medical Laboratory of Shenzhen Luohu People’s Hospital, Shenzhen, China; ^3^ The First Dongguan Affiliated Hospital of Guangdong Medical University, Dongguan, China

**Keywords:** enzyme, lysozyme, ZnO nanoparticles, nanomaterials, antibacterial activity

## Abstract

The lysozyme-modified nanoparticles (LY@ZnO NPs) were synthesized by the reduction–oxidation method, and the morphology and structure of LY@ZnO were analyzed by Fourier transform infrared (FTIR) spectroscopy, powder X-ray diffraction (XRD), scanning electron microsclope (SEM), and particle size analysis. The antibacterial effects of LY@ZnO against *Escherichia coli* (*E. coli*, Gram-negative bacteria) and *Staphylococcus aureus* (*S. aureus*, Gram-positive bacteria) were discussed by measuring the zone of inhibition (ZOI) and growth inhibition. The antimicrobial experiments showed that the LY@ZnO NPs possessed better antibacterial activity than ZnO. Besides, the antibacterial mechanism of LY@ZnO was also investigated, which was attributed to the generation of reactive oxygen species (ROS). Furthermore, the toxicities of LY@ZnO *in vivo* and *in vitro* were discussed by the cell counting kit-8 method and animal experiments, showing that LY@ZnO possessed excellent biocompatibility. Finally, the therapeutic effect of LY@ZnO on a rat skin infection model caused by methicillin-resistant *Staphylococcus aureus* (MRSA) was also studied, which exhibited good anti-infective activity. Our findings showed that LY@ZnO possessed remarkable antibacterial ability due to its excellent membrane permeability and small particle size. Besides, LY@ZnO also exhibited certain stability and great safety, which showed tremendous prospects for microbial infection in patients. It would also be helpful for a better understanding of the enzyme-modified nanomaterials against bacteria.

## Introduction

Bacterial infections have always been the main threat to human health all over the world (Deusenbery et al., 2021). According to the statistics from the World Health Organization (WHO), bacterial infections have been the leading cause of death in developing countries for the past decades (Eyvazi et al., 2020). However, the antibiotic-resistant bacteria (ARB) that are developed due to antibiotic drug abuse have significantly threatened public health. The production of ARB (especially superbug) has affected the therapeutic effect of traditional antibiotics (Theuretzbacher et al., 2020). So, researchers are dedicated to discover new drugs which can adequately inhibit bacterial growth and kill bacteria.

**GRAPHICAL ABSTRACT:**
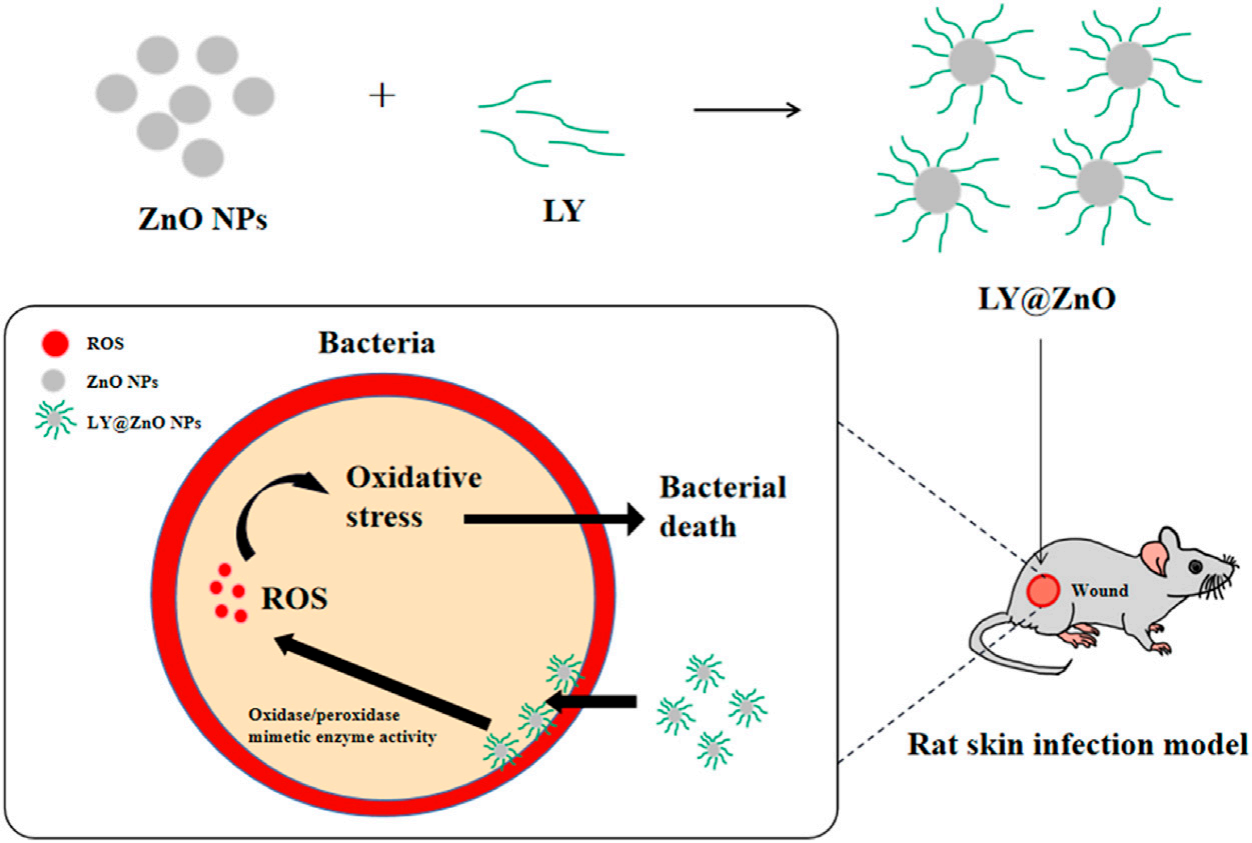


In the past decades, nanomaterials have achieved remarkable success in energy, catalysis, analysis, imaging, and diseases (Gao et al., 2004; Guo and Wang, 2011; Silvestre et al., 2011; Zhou et al., 2011; Jiang et al., 2012; Biju, 2013; Wang et al., 2020a; Rasal et al., 2021), particularly in the field of antibacterials (Xin et al., 2018). For example, inorganic nanomaterials (such as metal oxides) have attracted much attention due to their outstanding safety, stability, bio-distribution, and clearance (Chen et al., 2016; Guo et al., 2020; Wang et al., 2021a). Moreover, nanomaterials exhibited higher membrane permeability, which led to remarkable antibacterial ability, especially toward ARB (Han et al., 2017). Some literature reported that the nanomaterials could perform multiple bactericidal pathways, making it difficult for bacteria to adapt to these treatments, with great properties against ARB (Hajipour et al., 2012). Zinc oxide (ZnO, an essential metal oxide antibacterial) has aroused lots of attention because of its stability, bio-compatibility, and fast electron transfer kinetics (Du et al., 2021). Besides, ZnO NPs can damage the bacteria by the generation of the ROS, while the release of Zn ions leads to the dysfunction of critical components (such as DNA) (Qi et al., 2017). Hence, several studies on the antimicrobial properties of ZnO NPs are reported. For instance, Jian et al. investigated Ag nanoparticle-decorated ZnO nanorod arrays, which showed significantly high antibacterial properties against *Pseudomonas aeruginosa* (Shang et al., 20192019). Lan et al. found that the ZnO nanorods modified by the CeO_2_ NPs possessed antimicrobial capability toward the selected bacteria (*E. coli* and *S. aureus*) z` (Lan et al., 2018). Gutha et al. synthesized a CS-ZnO–based hybrid antibacterial material that displayed significant antibacterial effects against *E. coli* and *S. aureus*, while the antimicrobial rate has reached 99% in 3 h (Gutha et al., 2017). However, the cytotoxicity and poor selectivity limited the application of namomaterials as antibacterial agents. Therefore, to improve the biocompatibility of nano antibacterial agents has become a major need in this field.

Besides, enzymes are used as antibacterial agents due to the high selectivity toward substrates and high reaction efficiency under mild reaction conditions (Wang et al., 2019a; Wang et al., 2019b; Wang et al., 2020b; Mei et al., 2021; Tang et al., 2021; Wei et al., 2021; Zhang et al., 2021; Zhao et al., 2021). Lysozyme (LY), a typical antibacterial hydrolase, has attracted many interests due to its non-toxicity, specificity to hydrolyze towards bacterial walls, and the enhancement of immunity (Wu et al., 2017). For example, Ye et al. prepared hydrogel-immobilized lysozyme as an antibacterial agent, showing inhibition efficiency as high as 99.4% (against *E. coli* and *B. subtilis*) and a high effective duration of up to 55 days (Ye et al., 2019). Xiao et al. invented a moisture balanced antibacterial dressing loaded with lysozyme, which could facilitate the wound of infected mice healing, proving the lysozyme dressing could prevent infection of bacteria, efficiently (Xiao et al., 2019). Wu et al. reported that the lysozyme could cure the otorhinolaryngological inflammatory diseases (such as acute pharyngitis, acute laryngitis, and acute otitis media) caused by bacterial infection, effectively (Wu et al., 2017). However, the application of the lysozyme is limited by its unstable structure. Therefore, the design of a novel nano antibacterial agent to improve the antibacterial activity, stability, and biocompatibility was a promising research. In this research, ZnO nano antibacterial agents modified by the lysozyme displayed the following advantages: 1) the usage of lysozyme to modify ZnO could improve the selectivity and safety of the nanomaterials; 2) the utilization of ZnO as nanocarriers could improve the stability to acid and heat of enzyme due to the inherent core material, shape, size, and surface chemistry of nanomaterial; and 3) ZnO nanoparticles modified by lysozyme could exhibit the synergistic antibacterial effects.

Moreover, available research demonstrated that the ZnO antimicrobial agents could present their antibacterial effects by the production of intracellular ROS (Anicˇić et al., 2018; De Meutter and Goormaghtigh, 2020). For example, Traian et al. investigated the Mn-doped ZnO nanoparticles, which could promote the intracellular reactive oxygen species (ROS) generation in NIH3T3 fibroblast cells (Popescu et al., 2020). Bikram et al. studied the controlled copper doping in ZnO nanoparticles, finding that the nanoparticles could elicit ROS generation in macrophages (Das et al., 2019). Furthermore, the ROS-induced antimicrobial activity of metallic nanomaterials is often related to their intrinsic oxidase and peroxidase-mimetic enzyme catalytic activity (Yin et al., 2016; Chen et al., 2018). Many nanomaterials could produce ROS via the activities of oxidase–mimetic enzyme and peroxidase–mimetic enzyme.

In this study, the ZnO NPs modified with the lysozyme were successfully synthesized, and the structure of LY@ZnO was investigated by FTIR, XRD, SEM, and particle size analysis. Then, the antibacterial effects of LY@ZnO (enzyme-modified nanoantibiotics) against *E. coli* and *S. aureus* were studied via the turbidimetric method and disk-diffusion methods and found that compared with other common antibacterial agents, LY@ZnO possessed a remarkably good antibacterial effect. Besides, LY@ZnO also displayed low toxicities toward normal cells after a series of cytotoxicity assays, while showing no harm to rat organs in tissue section experiments. Furthermore, the antibacterial ability of LY@ZnO *in vivo* was discussed by the rat trauma infection model. Finally, the antibacterial mechanism of LY@ZnO NPs against *E. coli* and *S. aureus* was also investigated.

## Materials and Methods

### Materials and Reagents

2′,7′-dichlorofluorescein diacetate (DCFHDA) and zinc nitrate hexahydrate [Zn(NO_3_)_2_·6H_2_O] were bought from Shanghai Solarbio Co. Ltd. N-acetylcysteine (NAC) was obtained from Guangdong Macklin Biochemical Co. Ltd. LB agar powder and Reactive Oxygen Species Assay Kit were obtained through Dongguan Sigma-Aldrich. Gentamycin was bought from Guangzhou Macklin Co. Ltd. Yeast extract and tryptone were obtained from Shenzhen Macklin Biochemical Co. Ltd. Lysozyme was obtained from English Oxoid Corporation, and the sodium chlorides (NaCl) were bought from Shenzhen Solarbio Science and Technology Co. Ltd. *E. coli* (ATCC52922) and *S. aureus* (ATCC25923) were obtained from Zhanjiang Damao Co. Ltd. In this study, all chemical reagents were used at analytical grade, meaning that the reagents cannot be purified further.

The images of LY@ZnO were captured by Japan JEOL JEM-2100 Transmission Electron Microscope. The fluorescence spectroscopy was recorded by Japan Shimadzu RF-5301PC. The ultraviolet spectroscopy was studied by Thermo Fisher. The absorbance was obtained by Thermo Fisher’s Biomate 3S enzymatic labels. Zeta potential was measured by Nano ZS Zetasizer 90.

### Synthesis of LY@ZnO

The LY@ZnO NPs were obtained via the oxidation–reduction method (Cao et al., 2011). First, 50 ml LY solution (5.0 × 10^−5^ mol/L) was mixed with 50 ml Zn(NO_3_)_2_ (7.5 × 10^−2^ mol/L) in a 100-ml flask. The mixture was maintained for 24 h for the formation of the solution with LY-Zn^2+^ complexes. Then, 25 ml NaBH_4_ (0.10 mol/L) was added into the flask with vigorous stirring. Besides, the solution was kept under room temperature for 12 h with the protection of N_2_. Afterward, the samples would be separated *via* centrifugation at 10,000 rpm for 10 min. The collected samples were washed by anhydrous ethanol and vacuum dried for 24 h. Finally, the samples were calcinated under 80°C for 6 h. The LY-conjugated ZnO NPs (white powders) were gained.

### Antibacterial Experiment

#### Evaluation of Antibacterial Activity

The antibacterial ability of LY@ZnO was studied against two common clinical bacteria (*E. coli* and *S. aureus*) by turbidimetric method, while the minimal inhibitory concentration (MIC) of LY@ZnO was revealed. The LB medium was made of 10 g/L sodium chloride, 5 g/L yeast extract, and 10 g/L tryptone. Besides, 1.0 × 10^7^ CFU/ml of bacterial suspensions were inoculated into 2 ml of LB medium (additionally containing 20 mg/L of Zn(NO_3_)_2_, ZnO NPs, LY, and LY@ZnO). Then, the antibacterial efficacy would be calculated by directing the measurement of scattered light from samples.

The inhibition zone of the samples [Zn(NO_3_)_2_, ZnO NPs, LY, LY@ZnO, and gentamycin] were tested by the disk diffusion method, which indicated the antibacterial efficiency of the samples to be tested. First, 20 ml of LB medium was poured onto the sterilized plates until it solidifies horizontally. Then, the 0.1 ml strains were injected on the LB medium, which was coated. In addition, the filter planes with 15 μL respective sample solutions were added on the agar plate. The plates permeated with the equivalent LB liquids were regarded as the negative control. The dishes would be cultured at 37°C for 12 h, and the diameter of ZOI was measured. All experiments were duplicated three times under the same conditions.

#### Bactericidal Kinetics

To further evaluate the antibacterial activity of LY@ZnO, the time–disinfection kinetic curve was investigated under LY@ZnO treatment at different time points. The bacterial suspension (1.0 × 10^5^ CFU/ml) should be added into the different concentrations of LY@ZnO, which would be calculated with an oscillator at 37°C for 24 h. Then, 100 μL samples were diluted and coated on the LB medium, while the bacteria cultured for 0, 1, 3, 6, 12, 18, and 24 h would be gathered, respectively. The number of colonies was counted after the collected bacteria were cultured for 24 h.

#### Measurement of ROS

The ROS within the bacteria was tested via the Reactive Oxygen Species Assay Kit from Beyotime (Pinegin et al., 2017). First, 1.0 × 10^8^ CFU/ml of bacteria mixed with LY@ZnO would be centrifuged and gathered after culturing for 2 h at 37°C. After being washed with sterile water three times, the sample would be processed by 2′,7′-dichlorofluorescein diacetate after light-protection incubation for 3 h under room temperature. Then, the fluorescence intensity was measured by the Shimadzu RF-5301 PC Fluorescence Spectrophotometer, while the excitation wavelength was 488 nm and emission wavelength was 525 nm, respectively.

#### Activity of Oxidase Mimetic Enzymes

To evaluate the activity of oxidase mimetic enzymes, LY@ZnO was added into H_2_O_2_ from the Hydrogen Peroxide Assay Kit. The concentration of H_2_O_2_ would be measured according to the formula c = A/(*ε*×b) (c: concentration, A: absorbance, *ε*: molar extinction coefficient, b: light course). In this experiment, the absorbance was tested by NanoDrop 2000C (*ε* = 43.6 M^−1^ cm^−1^, b = 1 cm). The specific assay procedure should follow the instructions of the kit.

#### Activity of the Peroxidase-Mimicking Enzyme

To evaluate the activity of the oxidase mimetic enzymes, mixed solutions (containing 20 μL 100 mM of 3, 3′, 5, 5'–tetramethylbenzidine and 4 μL 10 M of H_2_O_2_) were added into 2 ml of LY@ZnO. Then, the absorbance was tested by the NanoDrop 2000C at different time points under different concentrations of LY@ZnO.

### Cytotoxicity

#### Toxicity Toward Cells

To evaluate the cytotoxicity of LY@ZnO toward mammalian cells, the viability of the cells (human normal hepatocytes: LO2, human normal lung epithelial cells: BEAS-2B, mouse neural cells: CATH.a, rat cardiomyocytes: H9C2, and rhinoceros fetal kidney cells: MA104) was revealed using the Cell Counting Kit-8 methods (CCK8). All cells were purchased from the Wuhan Procell Life Science and Technology Co., Ltd., which were cultured at 37°C with 5% CO_2_ in DMEM, which was complemented with 10% FBS. The LO2, BEAS-2B, CATH.a, H9C2, and MA104 cells were inoculated with 100 μL per well in 96-well plates at a density of 1 × 10^4^ cells/mL, respectively, while the 96-well plates would be cultured under 5% CO_2_ at 37°C for 12 h. Then, 200 μL DMEM (containing 10% FBS and 0, 5, 10, and 20 mg/L of LY@ZnO) was added into each well for 24 h after washing with PBS for 3 times, and five parallel groups should be set up for each concentration gradient. Afterward, 10 μL CCK8 was put into the well and then cultured at 37°C with 5% CO_2_ for 2 h. The UV absorption intensity of 96-well plates was measured by an enzyme marker at 450 nm. Cell survival rate (%) = [(As-Ab)/(Ac-Ab)] × 100% (As: experiential wells, Ab: blank wells with DMEM but no cells, Ac: control wells with cells but no LY@ZnO).

#### Tissue Section Observation

The 4-week-old SD female rats were purchased from the Guangdong Animal Centre, while the breeding of the rats and the animal experiments were processed in the Guangdong Medical University Laboratory Animal Centre. The 200 μL 20 mg/L of LY@ZnO was intraperitoneally injected into the rats, while the sanitary saline was regarded as the control group. The heart, liver, spleen, lungs, and kidneys of rats would be extracted after processing, which was immersed in 4% formalin. The sample would be stained by hematoxylin–eosin staining method after the paraffin section. Then, the slices were observed under an optical microscope.

#### Rat Infection Model

To investigate the antibacterial ability of LY@ZnO *in vivo*, 200 μL 1.0 × 10^9^ CFU/ml of MRSA was subcutaneously injected on the wounds of the infected rats. The 100 μL 10 mg/L of LY@ZnO was injected into rats by intraperitoneal injection, while the 100 μL of sanitary saline was used as the negative group and the 100 μL 10 mg/L gentamycin was used as the positive group. The size of the wounds was measured after 10 d of treatment. The bacteria were collected from the wounds, cultured in the LB medium, and the OD values were detected by the turbidimetric method.

## Results

### Characterization of LY@ZnO

**FIGURE 1:**
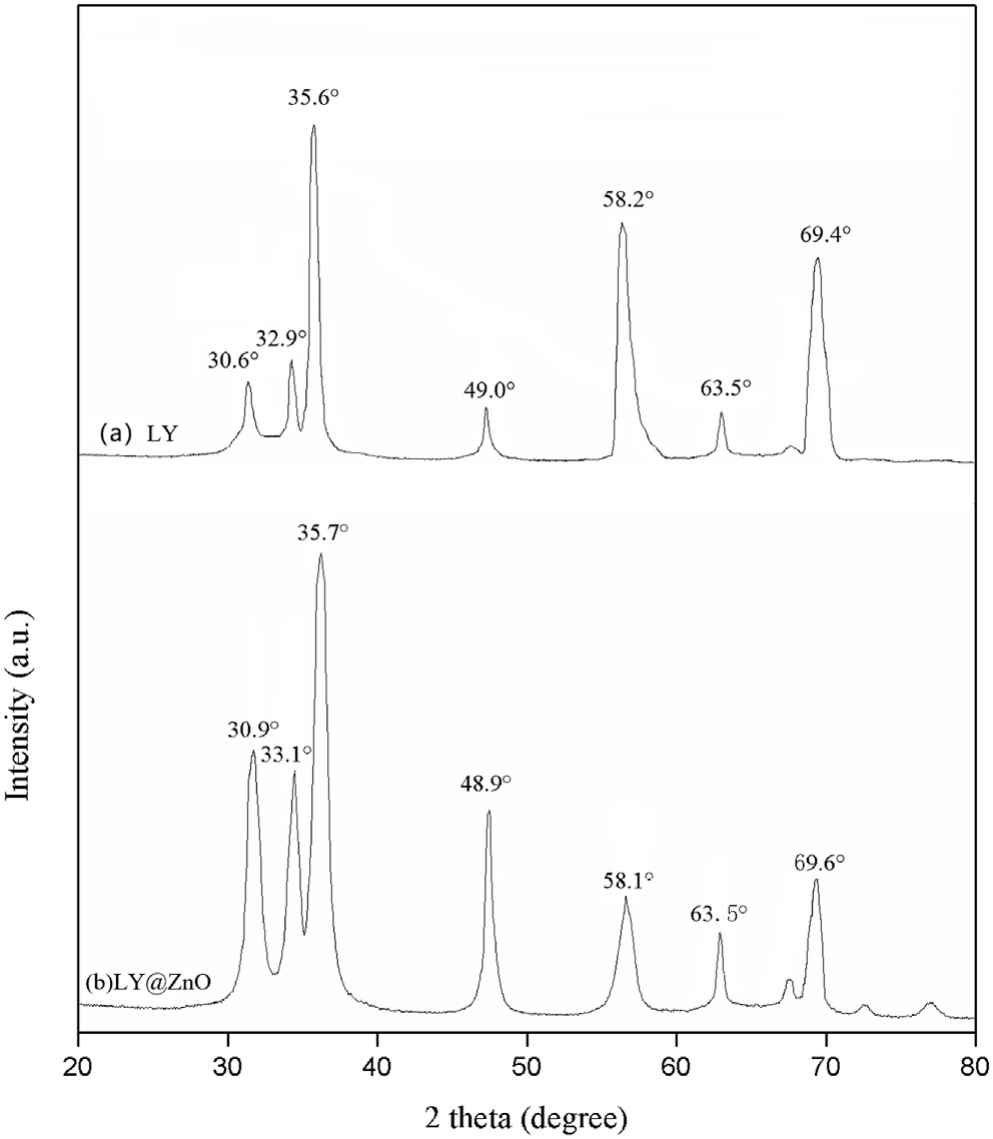
XRD spectrum of **(A)** ZnO and **(B)** LY@ZnO NPs.

Figure 1 shows the XRD spectrum of ZnO and LY@ZnO (LY-modified ZnO NPs). The characteristic diffraction peaks of 30.9°, 33.1°, 35.7°, 48.9°, 58.1°, 63.5°, and 69.6° were found to be the typical (100), (002), (101), (102), (110), (103), and (112) crystal peaks of ZnO NPs, respectively (JCPDS card: 36-1451). Besides, the crystal size (D) of LY@ZnO could be calculated by the Scherrer’s equation: D = k (*λ*/β cos *θ*) (k = 0.89, which is a constant; *λ* = 0.154 nm, which is the wavelength of the X-ray; *β* is the full width at half maximum; and *θ* = 27.2°, which is the half diffraction angle), and the average sizes (D) were calculated to be 11 nm.

**FIGURE 2:**
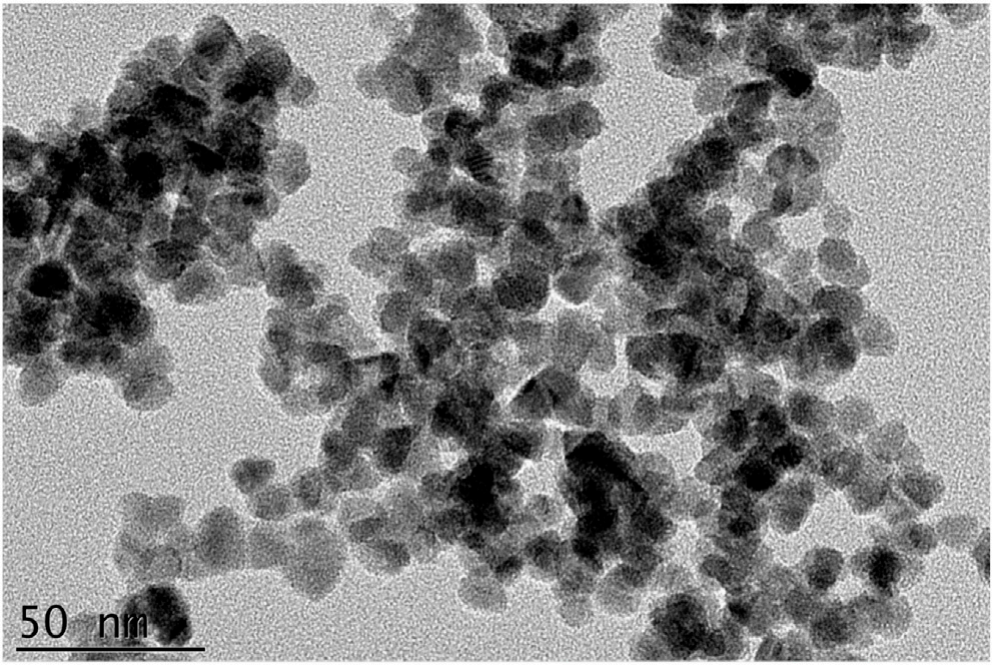
SEM image of LY@ZnO NPs.

**FIGURE 3:**
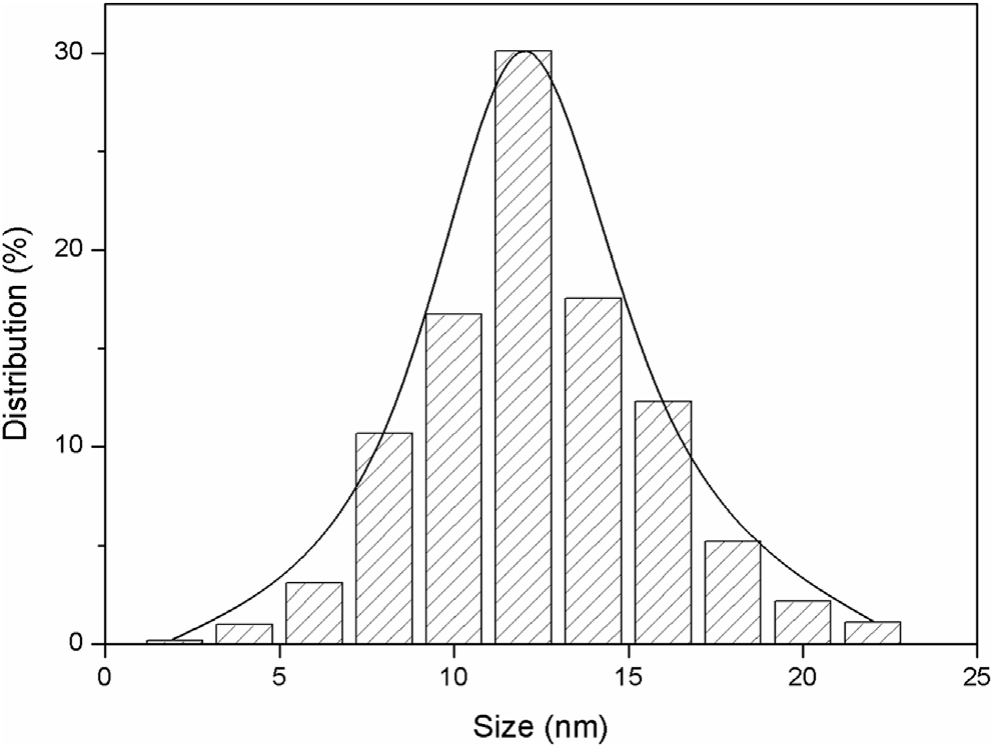
Nanoparticle size measurement result of LY@ZnO.

The TEM image of the LY@ZnO is presented in Figure 2. The NPs were the regular circles, and the average diameter was 12 nm (standard deviation values are calculated to be 1.09 from 10 samples). Figure 3 shows the nanoparticle dimensions of LY@ZnO tested by the nanoparticle size analyzer, and the nano size of LY@ZnO was determined to be about 12 nm. Therefore, the sizes of the LY@ZnO detected from the nanoparticle size analyzer were consistent with the TEM image and XRD analysis.

**FIGURE 4:**
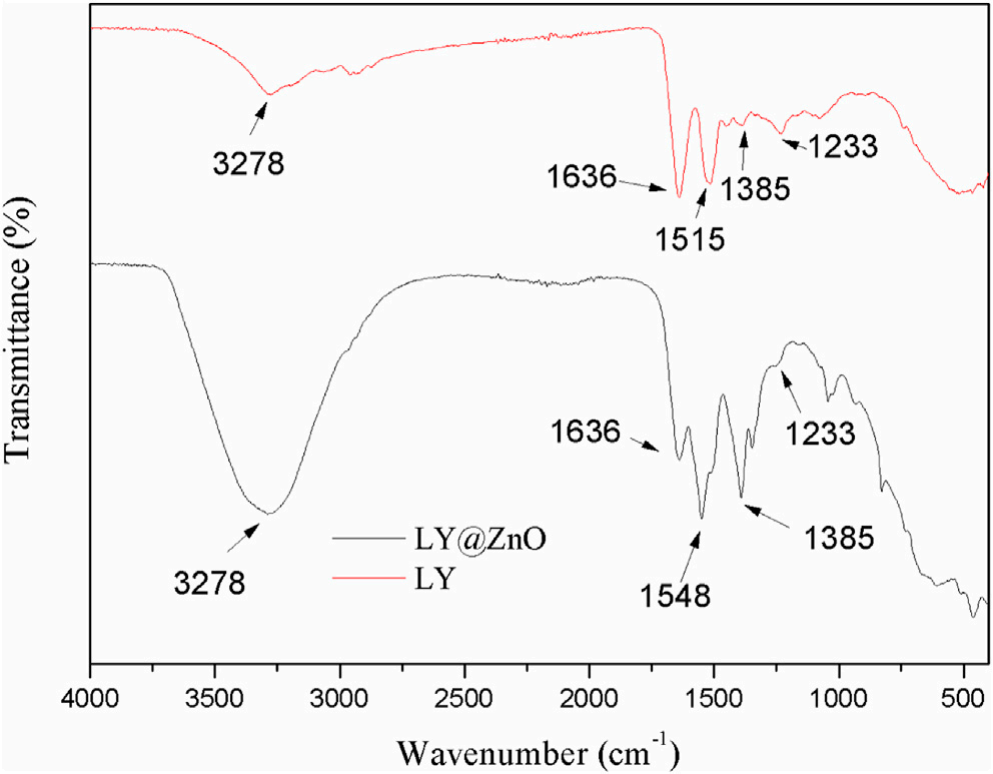
FTIR spectra of **(A)** LY and **(B)** LY@ZnO.

As shown in Figure 4, the LY and LY@ZnO NPs were analyzed via FTIR spectroscopy. The bands at 1,636 cm^−1^, 1,515 cm^−1^, 1,385 cm^−1^, and 1,233 cm^−1^ were found in the spectra of the LY (shown in Figure 4A). The peak at 1,636 cm^−1^ was generated from the C=O stretching vibration from the carboxyl group; the peak at 1,515 cm^−1^ was attributed to the N-H bending vibration in the amino group; the peak at 1,385 cm^−1^ was generated from the C-N stretching vibration in the amino group; the peak at 1,233 cm^−1^ was attributed to the C-S bending vibration in the sulfhydryl group (De Meutter and Goormaghtigh, 2020). After LY was conjugated with ZnO NPs, the absorption peaks of LY (the peaks at 1,636 cm^−1^, 1,385 cm^−1^, and 1,233 cm^−1^) were observed in the FTIR of LY@ZnO, which indicated that the structural integrity and functional groups of LY had been maintained during the modification process. Besides, the peaks at 1,515 cm^−1^ were shifted to 1520 cm^−1^ in the spectra of LY@ZnO compared with the FTIR spectra of LY, suggesting that LY was connected to ZnO via the strong interaction with the amino group of lysozymes.

### Antibacterial Activity

**FIGURE 5:**
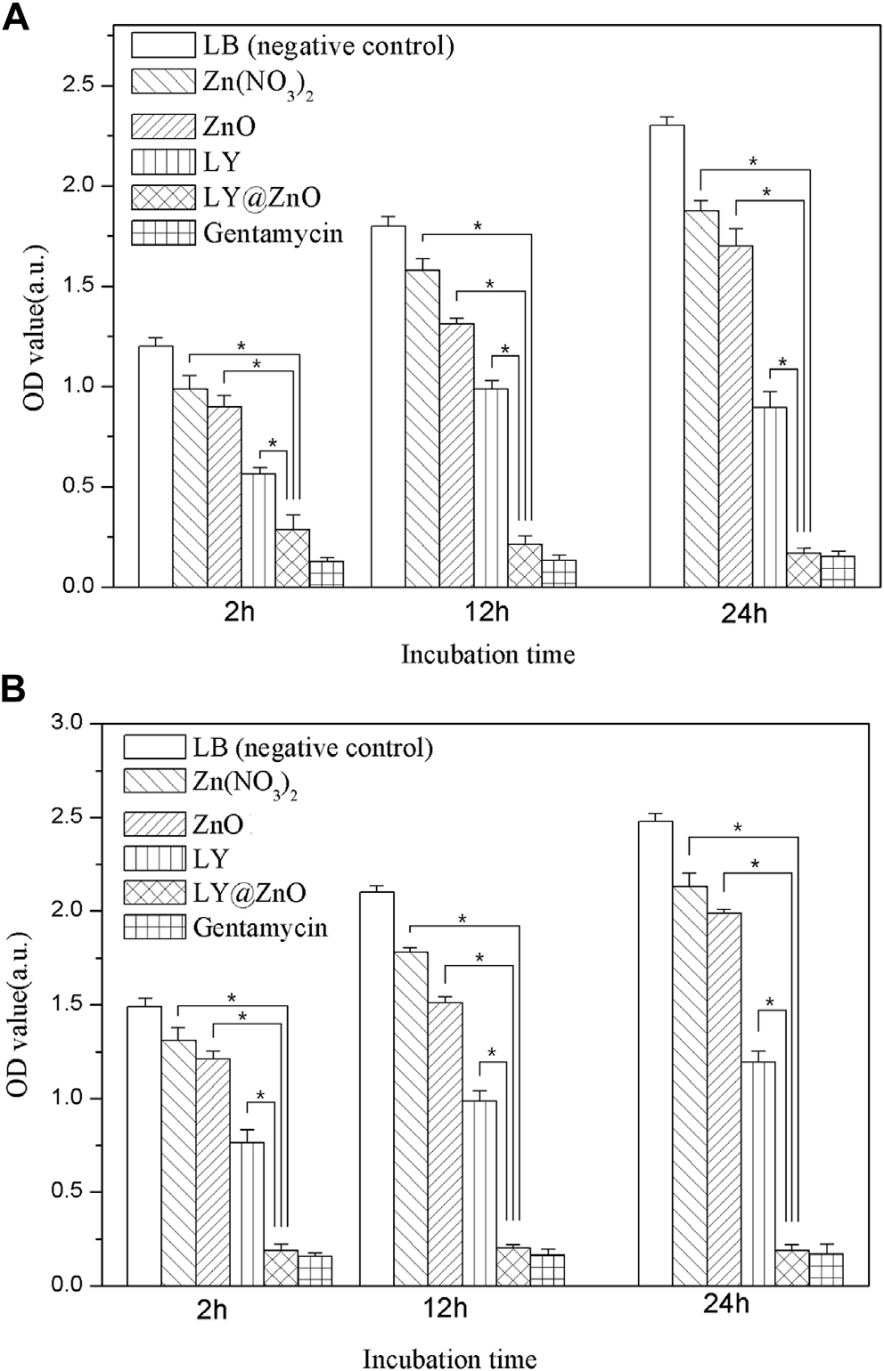
OD values of antibacterial activity for five antibacterial agents with the concentration of 20.0 mg/L observed to against bacteria. **(A)** *E. coli*; **(B)** *S. aureus*.

The antibacterial ability of LY@ZnO toward *E. coli* and *S. aureus* is revealed *via* the turbidimetric method (see in Figure 5). The results showed that the OD values (*E. coli* and *S. aureus*) were increased fast when the bacteria was cultured with LB medium (negative group), meaning that the negative group did not possess any inhibition against bacteria. In contrast, OD values were increased slowly when the bacteria were cultured in the positve group (gentamicin), meaning that gentamicin showed significantly good antimicrobial agents. Moreover, the OD values of LY@ZnO were slightly higher than the positive group, indicating that the antibacterial effects of LY@ZnO against microorganisms were prominent. Besides, the OD values of LY and ZnO were higher than those of the LY@ZnO group, suggesting that the ZnO NPs and the LY possessed a certain level of antibacterial effects. These results revealed that LY@ZnO was an outstanding antibacterial agent, and the antibacterial ability might be generated by the collaborative therapeutic effects of LY and ZnO.

**FIGURE 6:**
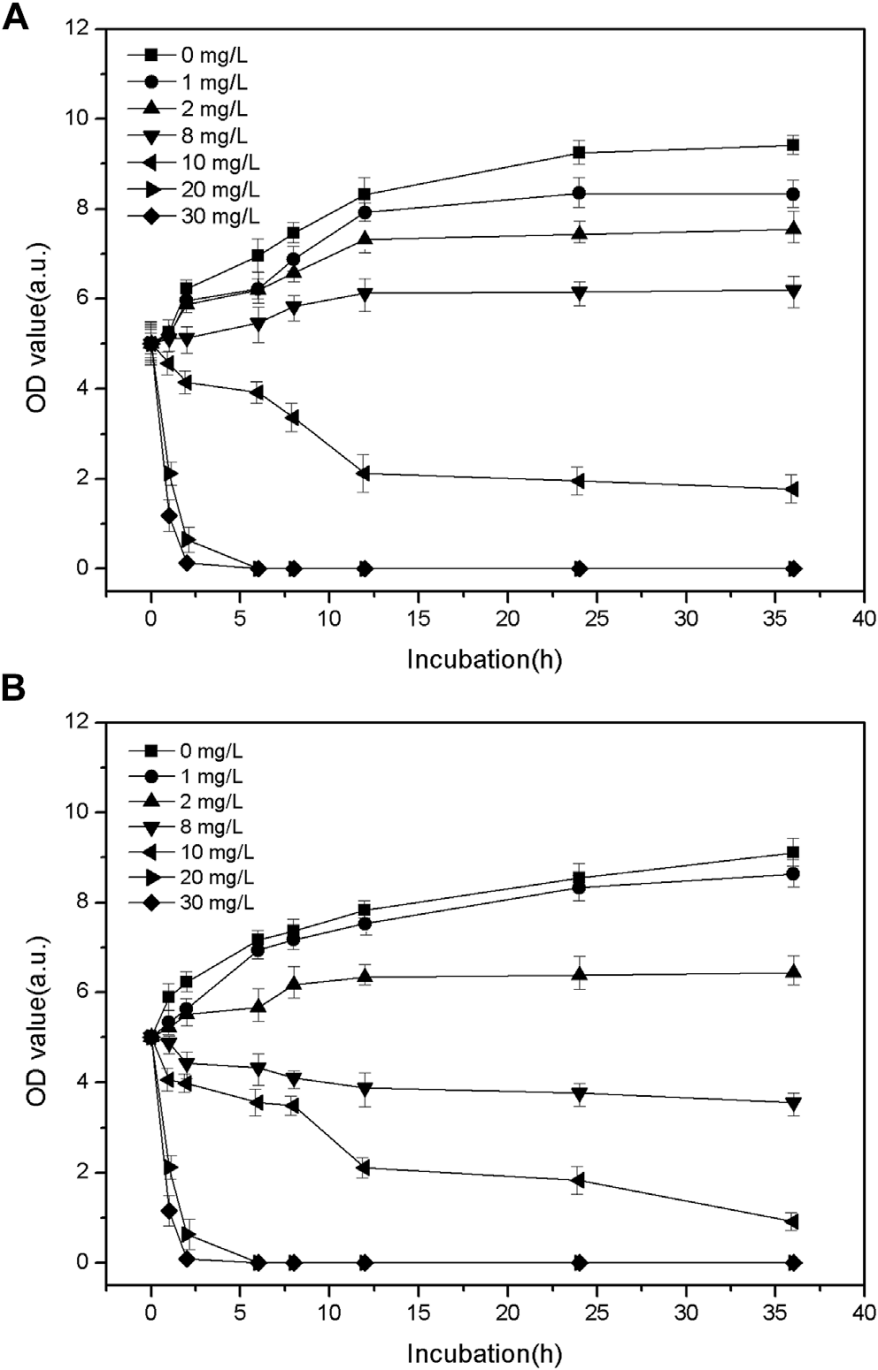
Bactericidal kinetics curves of LY@ZnO against **(A)** *E. coli* and **(B)** *S. aureus*.

The bactericidal kinetics characteristics of LY@ZnO are shown in Figure 6. The OD values were still increased when the bacteria was incubated with a low concentration of LY@ZnO (below 8.0 mg/L). However, the OD values decreased remarkably when the bacteria were cultured with high concentration of LY@ZnO, showing that the high concentration of LY@ZnO could kill both *E. coli* and *S. aureus* effectively. Furthermore, it was found that the OD values were nearly unchanged after 12-h incubation with LY@ZnO, which illustrated that the LY@ZnO NPs could completely inhibit the growth of *E. coli* and *S. aureus* after 12 h. The relatively rapid bactericidal kinetic profile further demonstrated its great potential as a nanoantibiotic.

**FIGURE 7:**
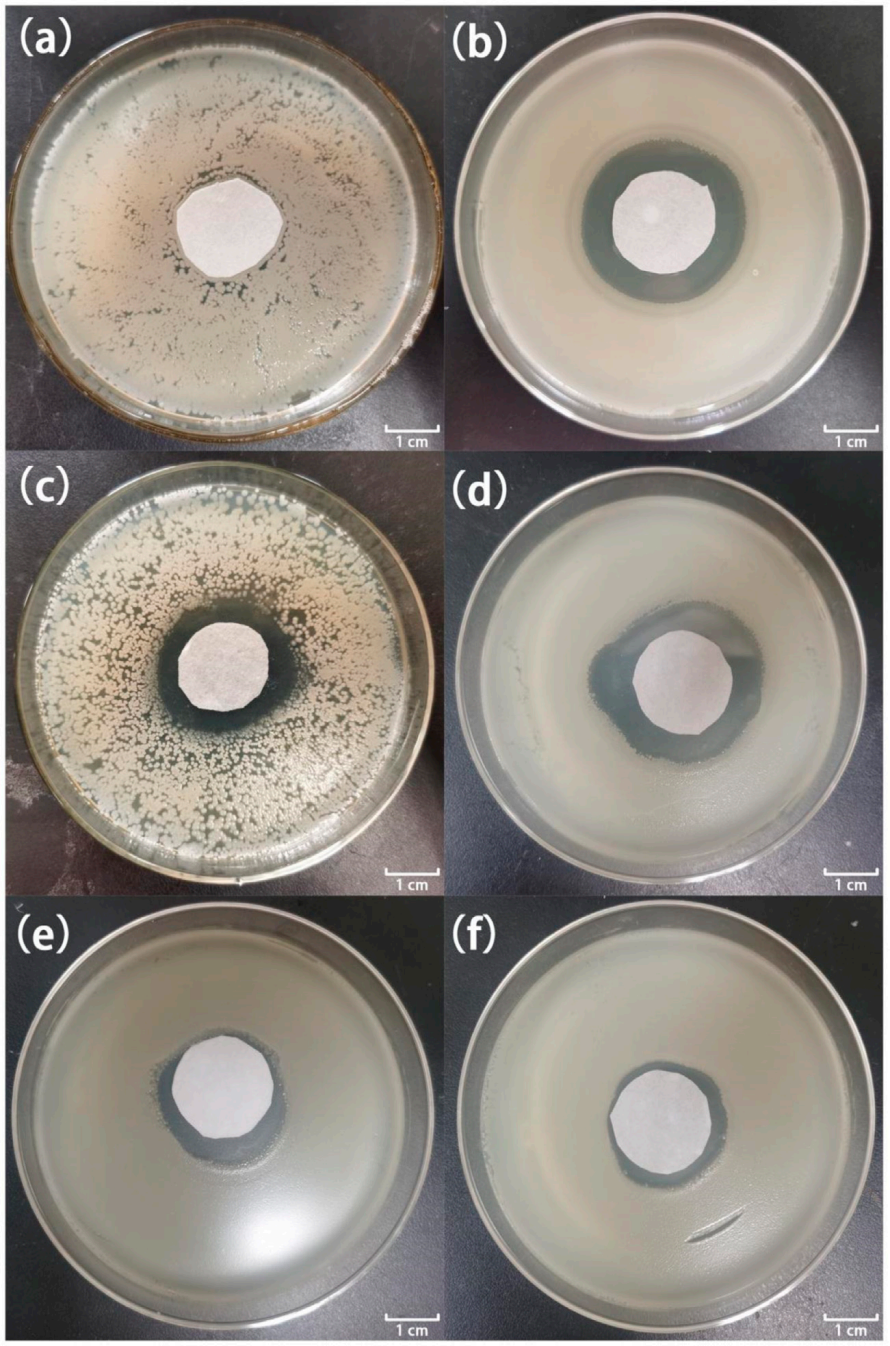
Images of inhibition zones for different samples against *E. coli*. **(A)** 0.0 μg; **(B)** 20.0 μg gentamycin; **(C)** 5.0 μg LY@ZnO; **(D)** 20.0 μg LY@ZnO; **(E)** 20.0 μg LY; and **(F)** 20.0 μg ZnO.

**FIGURE 8:**
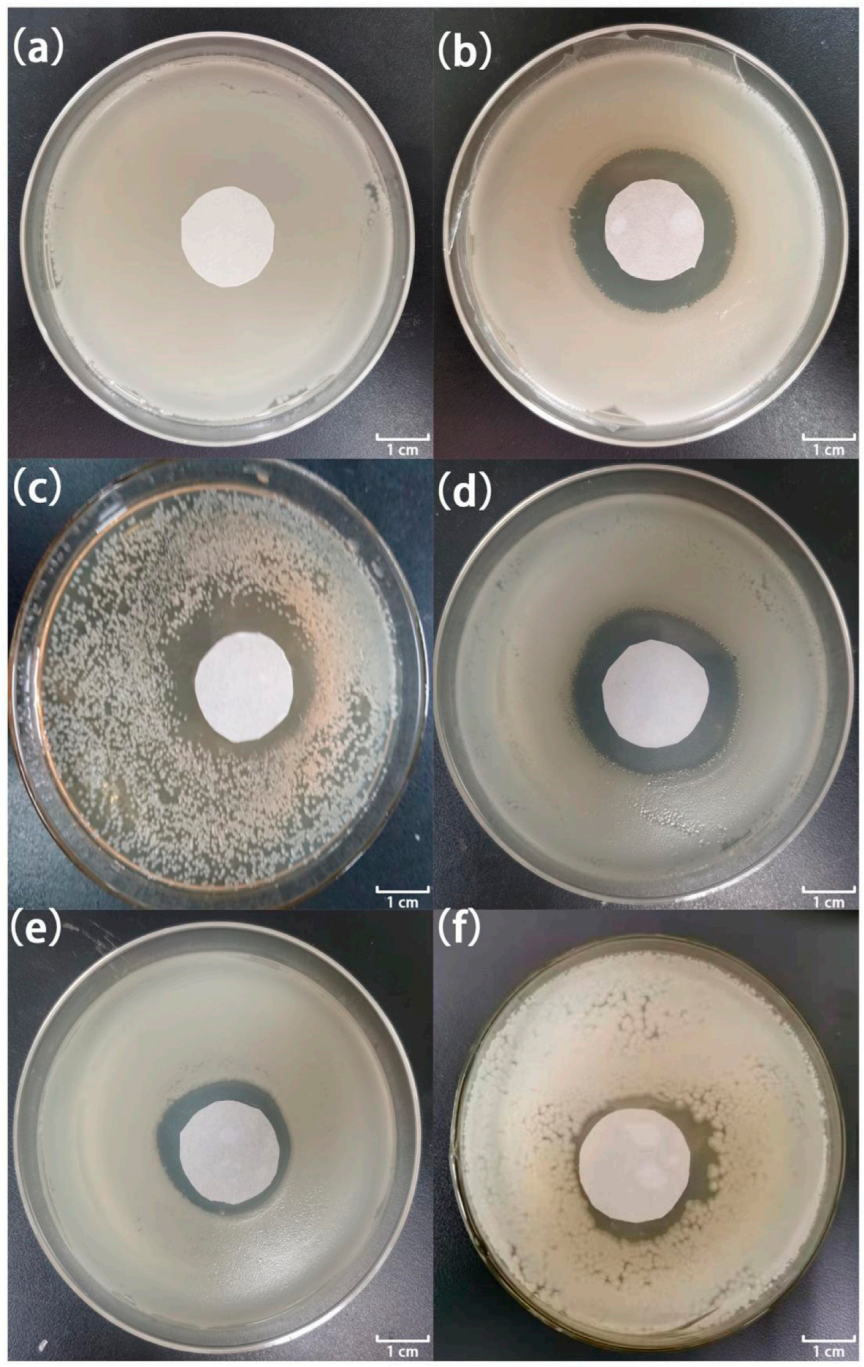
Images of inhibition zones for different samples against *S. aureus*. **(A)** 0.0 μg; **(B)** 20.0 μg gentamycin; **(C)** 5.0 μg LY@ZnO; **(D)** 20.0 μg LY@ZnO; **(E)** 20.0 μg LY; and **(F)** 20.0 μg ZnO.

Figures 7, 8 show the antibacterial effects of LY@ZnO against *E. coli* and *S. aureus* via disc diffusion methods. From the results shown in Figures 7A, 8A, we can observe that the negative groups displayed no ZOI, which indicated that the bacteria bred rapidly in the LB medium. Furthermore, the LY and ZnO groups showed small ZOI, exhibiting a certain level of antibacterial ability. Noticeably, as shown in Figures 7C,D, 8C,D, the ZOI expanded as the concentration of LY@ZnO increased, meaning that the inhibition of LY@ZnO was dependent on concentrations. Especially, as shown in Figures 7B,D, 8B,D, the diameter with high concentrations was almost the same as the diameter of the positive group (*E. coli* and *S. aureus*), which were consistent with the results of the turbidimetric method.

Moreover, the MICs of LY@ZnO against *E. coli*, *S. aureus*, *Streptococcus haemolyticus*, *Corynebacterium diphtheriae*, and *Legionella* were 2.0, 4.0, 8.0, 16.0, and 16.0 μg/ml, respectively. These results suggested that LY@ZnO was an excellent broad-spectrum antimicrobial agent.

### Antibacterial Mechanism

#### ROS Generation

**FIGURE 9:**
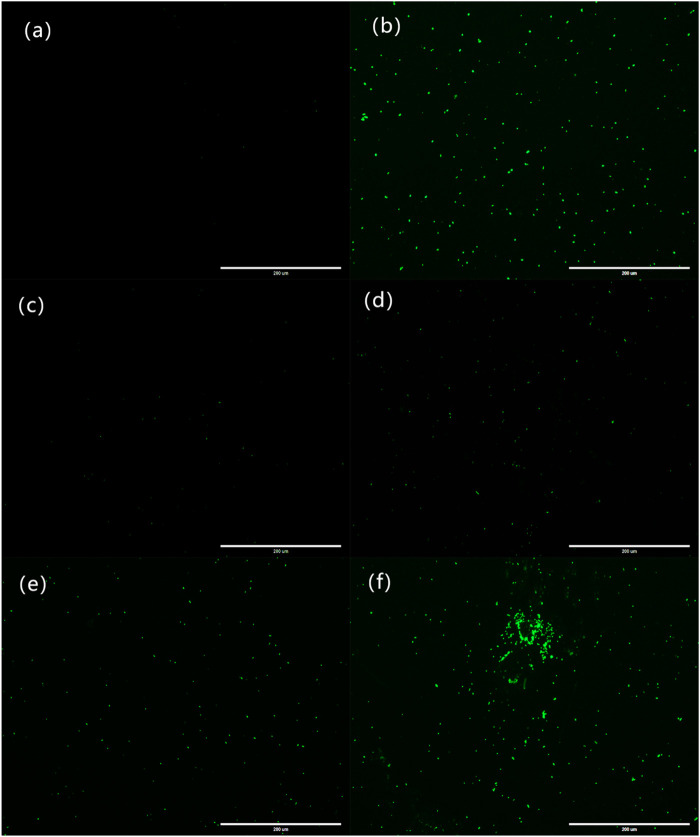
Laser confocal microscopy to observe the effect of LY@ZnO on ROS in bacteria. **(A)** 0.0 mg/L LY@ZnO; **(B)** 20.0 mg/L gentamycin; **(C)** 20.0 mg/L LY; **(D)** 20.0 mg/L ZnO; **(E)** 5.0 mg/L LY@ZnO; and **(F)** 20.0 mg/L LY@ZnO.

**FIGURE 10:**
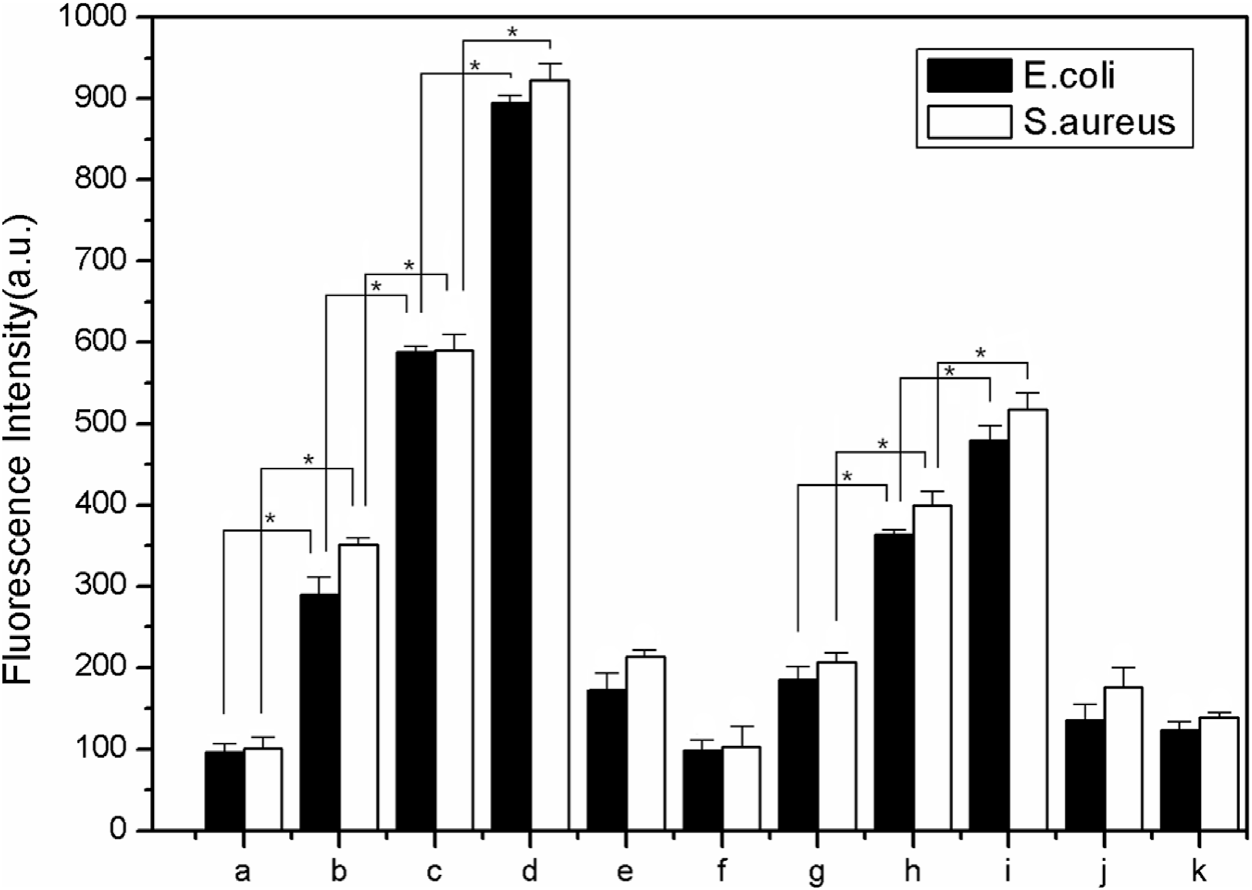
The qualitative measurement to determine the effect of antioxidant on ROS generation in *E. coli* and *S. aureus* during 24 h **(A)** 0.0 mg/L LY@ZnO;**(B)** 5.0 mg/L LY@ZnO; **(C)** 10.0 mg/L LY@ZnO; **(D)** 20.0 mg/L LY@ZnO;**(E)** 20.0 mg/L ZnO **(F)** 20.0 mg/L LY **(G)** 10.0 μg/L NAC + 20.0 mg/L LY@ZnO **(H)** 10.0 μg/L NAC + 20.0 mg/L LY; and **(I)** 10.0 μg/L NAC + 20.0 mg/L LY@ZnO **(J)** 10.0 μg/L NAC + 20.0 mg/L ZnO.

The fluorescence images of the bacterial ROS changes are presented in Figure 9, while the production of ROS is presented through the qualitative assessment of LY@ZnO in Figure 10. It can be found in Figure 9 that the fluorescence intensities turned stronger as the concentration of LY@ZnO increased, and these images suggested that the ROS generation was promoted when the LY@ZnO was presented. As shown in Figure 10A, the low level of fluorescence intensities belonged to the negative group (only bacteria exist). However, after being treated with LY@ZnO, the fluorescence intensities of *E. coli* and *S. aureus* were higher than those of the negative group, LY group, and ZnO group (see in Figures 10A,D–F), showing that more production of ROS resulted from the LY@ZnO group. Furthermore, In the presence of N-acetylcysteine (NAC), the antibacterial effects were also studied. NAC (an effective antioxidant) could prevent the oxidative damage due to the mercapto group of NAC. The fluorescence intensities were remarkably decreased when the NAC was added into 5.0, 10.0, and 20.0 mg/L of LY@ZnO, respectively (see in Figures 10G–I). These results clearly illustrated that the antimicrobial activities of LY@ZnO were decreasing in the presence of antioxidants and further explained that the intracellular ROS generation participated in the antibacterial process of LY@ZnO.

**FIGURE 11:**
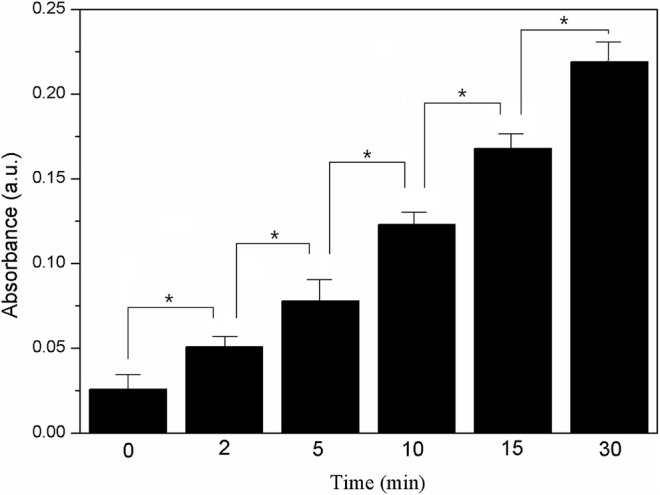
UV spectra of LY@ZnO mixed with TMB at different times.

**FIGURE 12:**
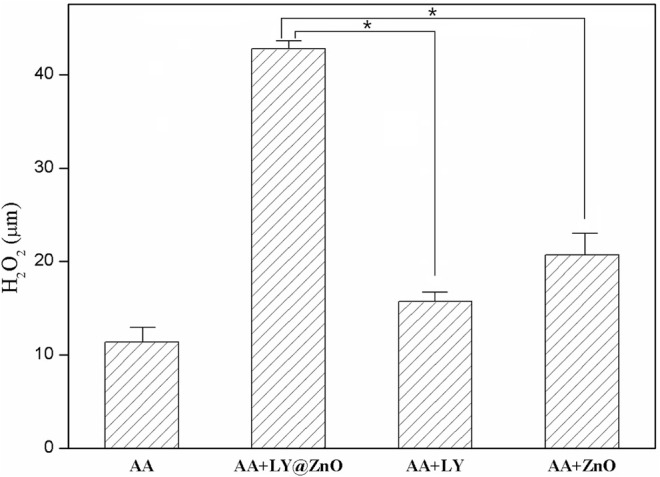
Concentration of H_2_O_2_ after treated with AA, AA + LY@ZnO, AA + LY, and AA + ZnO.

**FIGURE 13:**
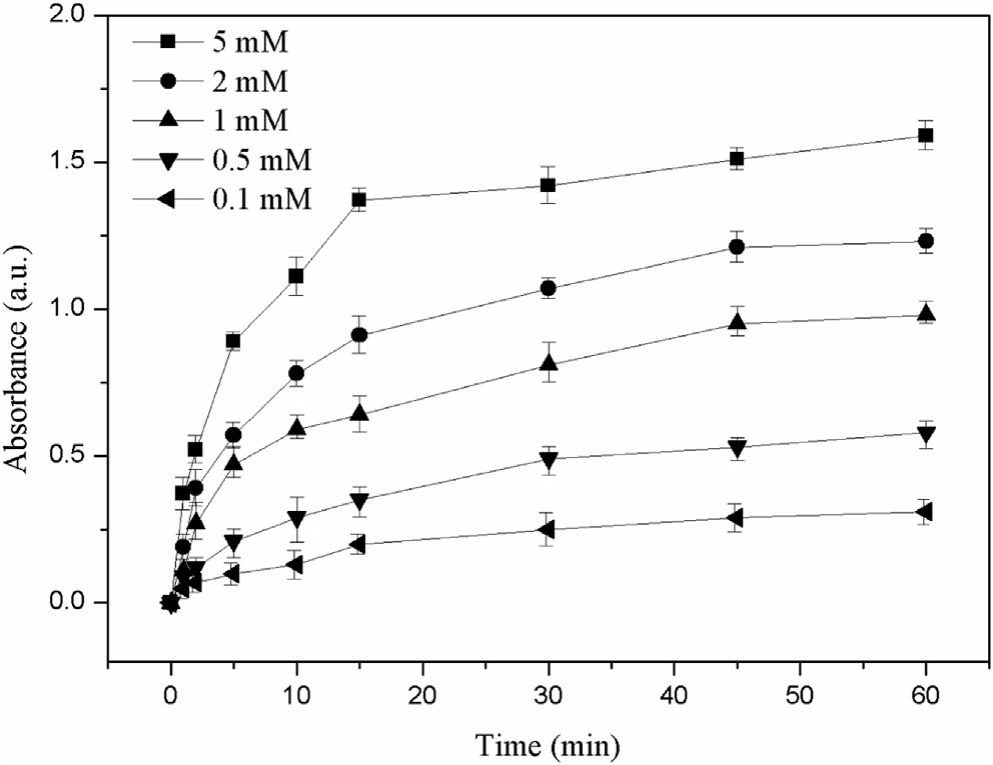
UV absorbance change of TMB (653 nm) with different concentrations of (mM).

Furthermore, it was known that the ROS-induced antimicrobial activity of precious metal nanomaterials was associated with their intrinsic oxidase and peroxidase–mimetic enzymatic catalytic activities (Tao et al., 2014; Anicˇić et al., 2018; Wang et al., 2021b). The oxidase–mimetic enzymatic activity of LY@ZnO was measured using TMB as a substrate. TMB appeared blue after oxidation and had a maximum UV absorption at 653 nm. As shown in Figure 11, the absorbance was increased since LY@ZnO mixed with TMB, indicating that that catalytic reaction started after the TMB mixed with LY@ZnO, and the catalytic efficiency was increased as the reaction time increased. Figure 12 showed the H_2_O_2_ concentration after being treated with ascorbic acid (common intracellular antioxidants, AA), AA + LY, AA + ZnO, and AA + LY@ZnO, respectively. After H_2_O_2_ was treated with AA, LY, ZnO, and LY@ZnO groups, the concentrations of H_2_O_2_ were low (see in Figure 12). However, after H_2_O_2_ was treated with the LY@ZnO group, the highest concentration of H_2_O_2_ was observed, indicating that the LY@ZnO possessed the remarkable activity of the oxidase mimetic enzymes. Moreover, TMB is a common substrate for peroxidase, which could be reduced by the peroxidase-mimicking enzyme. Figure 13 presents the absorbance of TMB treated with different concentrations of LY@ZnO. As shown in Figure 13, the absorbance showed concentration dependence as the concentration of LY@ZnO changed, meaning that LY@ZnO exhibited the activity of peroxidase-mimicking enzyme. Therefore, the generation of ROS might be due to the inherent oxidase–mimetic and peroxidase–mimetic enzyme activity of LY@ZnO.

### Biocompatibility

#### CCK8 of the Normal Cells

**FIGURE 14:**
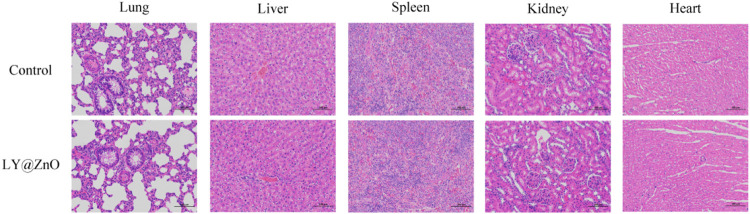
Cell viability of LO2, BEAS-2B, CATH.a, H9C2, and MA104 with the treatment of 0.0, 5.0, 10.0, and 20.0 mg/L LY@ZnO, 20.0 mg/L LY, and 20.0 mg/L ZnO.

Based on the LO2, BEAS-2B, CATH.a, H9C2, and MA104 cells as the cellular toxicity models, the toxicity against cells is shown in Figure 14. The cell viability of LO2, BEAS-2B, CATH.a, H9C2, and MA104 were 86.2%, 89.8%, 88.4%, 91.0%, and 80.7%, respectively, with the concentration of 20.0 mg/L LY@ZnO, which proved that the LY@ZnO showed low toxicity against normal cells. However, the cell viability of gentamycin was lower than that of the LY@ZnO groups at the same concentration, showing that LY@ZnO might be a safer antibacterial agent than gentamycin.

#### Pathological Sections

**FIGURE 15:**
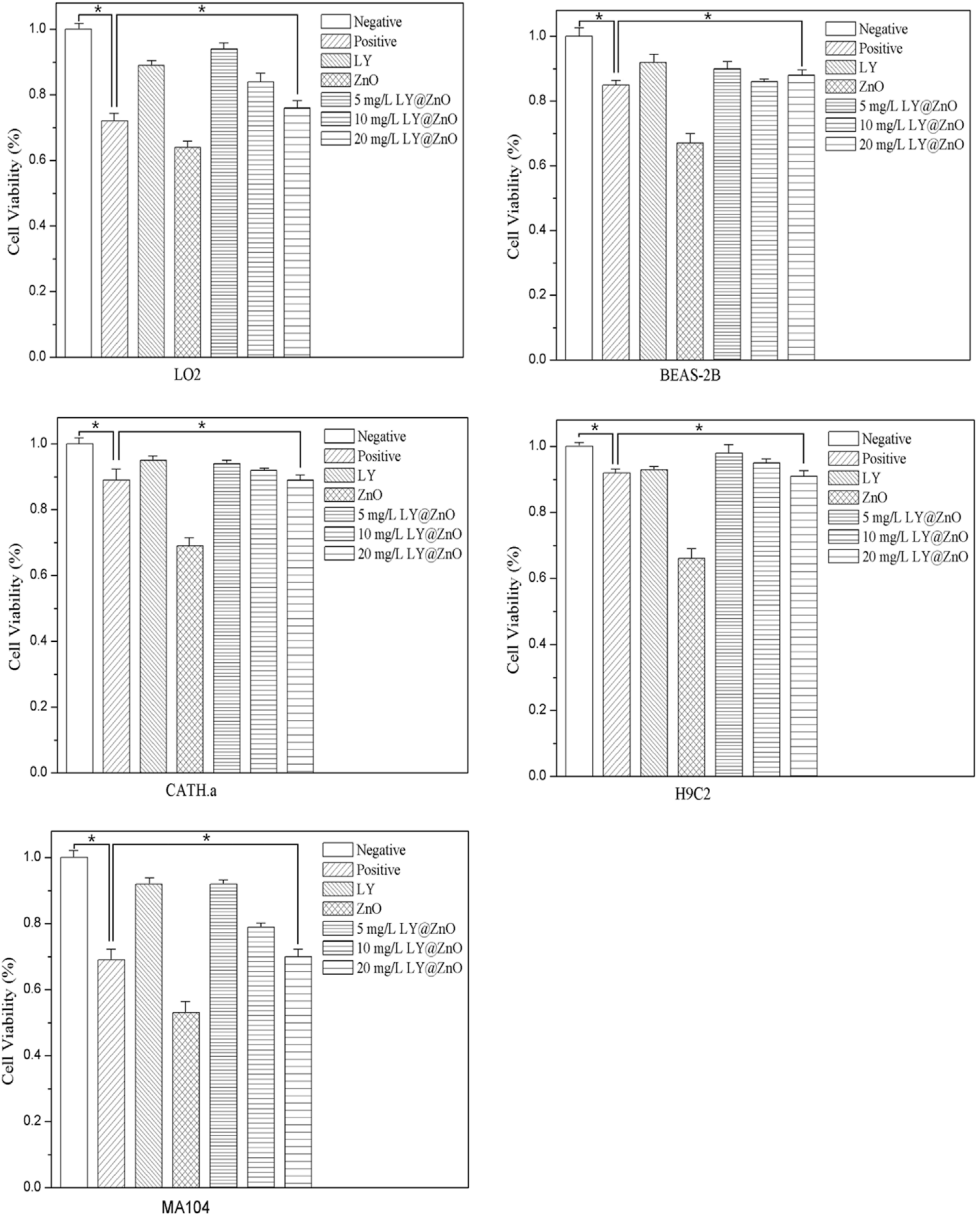
Tissue section of the lung, liver, spleen, kidney, and heart with 20.0 mg/L LY@ZnO processed.

The pathological sections of the lung, liver, spleen, kidney, and heart treated with LY@ZnO are presented in Figure 15. Compared with the negative group (physiological saline group), the LY@ZnO group showed no clear damage to the rat organs, indicating that LY@ZnO possessed low toxicity and excellent biocompatibility *in vivo*. These experiments displayed that LY@ZnO had a great prospect in the treatment of bacterial infection *in vivo*.

### *In vivo* Antimicrobial Properties

**FIGURE 16:**
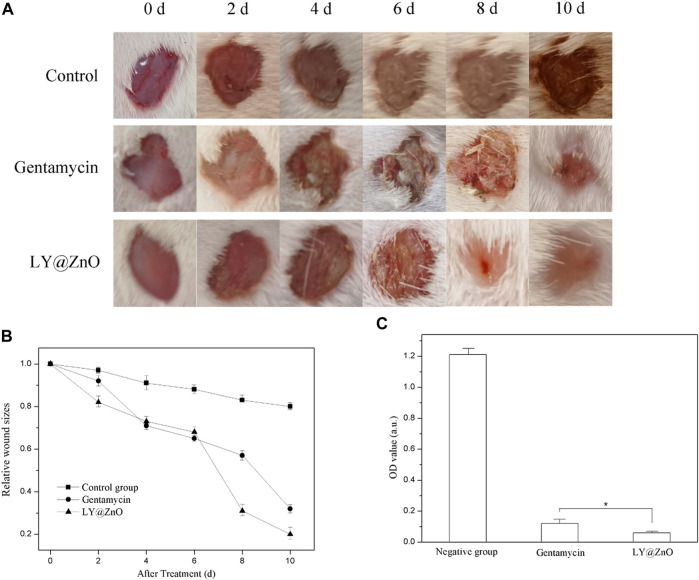
**(A)** Images of wounds with sanitary saline, gentamycin, and LY@ZnO injected at different time points, **(B)** the size of wounds at different time points, **(C)** the OD value of bacteria obtained from the wound after 2 h cultured.

In our experiments, the rat skin infection model was used to estimate the treatment of LY@ZnO against the MRSA *in vivo* (see in Figure 16). As shown in Figure 16A, the infected wound healing in the negative group was poor. However, compared with the negative group, the infected wounds in the LY@ZnO were basically recovered, showing good efficacy in the treatment of the infected wounds. Furthermore, the relative wound sizes are shown in Figure 16B. It was found that the relative wound size of the LY@ZnO group was smaller than that of the positive group and the negative group. These results indicated that LY@ZnO could facilitate the healing of infected wounds. Besides, the OD values of bacteria obtained from the wound are shown in Figure 16C. The OD values of the LY@ZnO group were lower than those of the positive and the negative groups, showing excellent therapeutic effects against infected wounds caused by bacteria *in vivo*. Therefore, the LY@ZnO possessed great potential for clinical application in treating infectious diseases, especially infections induced by multidrug-resistant bacteria.

## Conclusion

In conclusion, the LY@ZnO NPs were synthesized successfully by the reduction–oxidation method. After a series of antibacterial experiments tested, the high antibacterial ability against *E. coli* and *S. aureu*s of LY@ZnO were proposed, which might be attributed to the synergistic effects by LY and ZnO nanoparticles. The antibacterial mechanism of LY@ZnO was discovered to induce the death of bacteria by ROS generation. The antibacterial mechanism of LY@ZnO was discovered to induce the death of bacteria by ROS generation. The generation of ROS might be attributed to the oxidase–mimetic/peroxidase–mimetic enzyme activity of LY@ZnO. Moreover, the biological effect of the LY@ZnO *in vitro* and *in vivo* was evaluated, which showed low toxicity, excellent biocompatibility, and facilitating the healing. Our findings revealed that LY@ZnO showed fascinating prospects as an antibacterial drug, which provided clinical application in the field of wound anti-infection therapy.

## Data Availability

The original contributions presented in the study are included in the article/Supplementary Material; further inquiries can be directed to the corresponding authors.
